# Case report: Saccadic ping-pong gaze in progressive supranuclear palsy with predominant postural instability

**DOI:** 10.3389/fneur.2023.1100931

**Published:** 2023-03-01

**Authors:** Hikari Nunomura, Taketoshi Kasahara, Taku Hatano, Hitoshi Shimada, Yuhei Takado, Hironobu Endo, Ayako Inoshita, Atsuko Inomata, Toshihisa Murofushi, Shihoko Misawa, Yutaka Machida, Hisamasa Imai

**Affiliations:** ^1^Department of Neurology, Tokyo Rinkai Hospital, Tokyo, Japan; ^2^Division of Rehabilitation, Tokyo Rinkai Hospital, Tokyo, Japan; ^3^Department of Neurology, School of Medicine, Juntendo University, Tokyo, Japan; ^4^Department of Functional Neurology & Neurosurgery, Center for Integrated Human Brain Science, Niigata University, Niigata, Japan; ^5^Department of Functional Brain Imaging Research, Institute for Quantum Medical Science, Quantum Life and Medical Science Directorate, National Institutes for Quantum Science and Technology (QST), Chiba, Japan; ^6^Department of Otorhinolaryngology, School of Medicine, Juntendo University, Tokyo, Japan; ^7^Department of Otorhinolaryngology, Mizonokuchi Hospital, Teikyo University, Kawasaki, Japan

**Keywords:** progressive supranuclear palsy, postural instability, macro square wave jerks, ping-pong gaze, saccadic ping-pong gaze, transitory alternating saccades, the movement disorder society criteria, case report

## Abstract

We report a 63-year-old female patient with progressive supranuclear palsy (PSP). She presented predominant postural instability and “saccadic ping-pong gaze” (SPPG). She had unprovoked falls recurrently within a year from the onset of gait disturbance. She tended to fall backward with eye closure but had no freezing of gait on examination. She showed no signs of nuchal dystonia, limb tremor, rigidity, spasticity, or ataxia. The dopaminergic response was negative. On the initial examination, her vertical eye movements were normal, but frequent macro square wave jerks and SPPG were observed. SPPG consisted of short-cycle, horizontal conjugate irregular pendular oscillations of the eye position from the midpoint with superimposed small saccades. SPPG was observed usually in the dark, not in the daylight, and with eye closure by using electrooculogram and infrared charge-coupled device imaging. One and a half years after the first examination, she was diagnosed as probable PSP with vertical supranuclear gaze palsy. SPPG was first described in patients who are unconscious by Johkura in 1998 as a “saccadic” variant of “ping-pong gaze (PPG).” PPG, short-cycle periodic alternating gaze, has been described in comatose patients since 1967. On the other hand, abnormal eye movement, which looks the same as SPPG in coma, has been described in conscious patients with PSP or spinocerebellar degeneration (SCD) in Japanese literature since 1975. However, it has been called “transient alternating saccades (TAS).” Nowadays, we believe it is more appropriate to call this abnormal eye movement “SPPG” instead of TAS. Here, we propose that PSP, a neuro-degenerative disease, should be added as one of the etiologies of SPPG. We discuss the differences between PPG/SPPG in coma and SPPG in PSP and the possible pathophysiological mechanism of SPPG in relation to cerebellar oculomotor dysfunctions.

## Introduction

“Saccadic ping-pong gaze (SPPG),” a rare and unusual disorder, was first reported in patients who are unconscious by Johkura et al. (1) as a saccadic variant of “ping-pong gaze (PPG),” horizontal conjugate short-cycle periodic alternating gaze. In 1967, Fisher (2) first gave a brief description of PPG in a comatose patient. The term PPG was first used in 1979. Here, we first report “SPPG” in a conscious patient with progressive supranuclear palsy (PSP). This SPPG was observed with electrooculogram (EOG) and infrared charge-coupled device (CCD) imaging in the dark and with eye closure. The same abnormal eye movement in a conscious patient, as we report here, was first described by Komatsuzaki and Mizutani (3) in a patient with spinocerebellar degeneration (SCD) in 1975. They named it “transitory alternating saccades (TAS).” To date, all cases with TAS in PSP or SCD have been reported in Japanese (not in English), although some have English abstracts. SPPG in the present patient and so-called TAS had essentially the same waveform as SPPG in comatose patients. However, SPPG in the present patient occurred in quite different conditions and had several different features from PPG/SPPG in coma. We detail SPPG in PSP and discuss the clinical and pathophysiological implications.

## Case description

Our patient, a 63-year-old woman, began to have difficulty walking 5 years before presentation. She experienced unprovoked falls recurrently within a year from the onset of her gait disturbance. She became unable to walk by herself after 3 years from the onset. Treatment of levodopa/carbidopa (750 mg/75 mg) was ineffective. On the initial examination, she had normal cognition but had signs of mild bradyphrenia and slurred speech. Her horizontal and vertical eye movements were normal, but there appeared impaired convergence and horizontal square wave jerks (SWJs). She showed no signs of nuchal dystonia, limb tremor, rigidity, spasticity, or limb ataxia. In addition, she showed no signs of peripheral sensory or motor neuropathy. On the other hand, she showed severe disorders of posture and gait. She had a broad-based stance and tended to fall backward with eye closure and actually fell down on the pull backward test if not caught by the examiner. Her gait was wide-based and short-stepped and characterized by reduced rotation of the trunk. Although her gait was unstable, she had no freezing of gait (4) (see Supplementary Video). She was diagnosed as suggestive of PSP with predominant postural instability (5) by a combination of level 3 in oculomotor dysfunction (SWJs) and level 1 in postural instability (unprovoked falls) according to the Movement Disorder Society criteria 2017 (the MDS criteria) for PSP (6).

**Figure 1:**
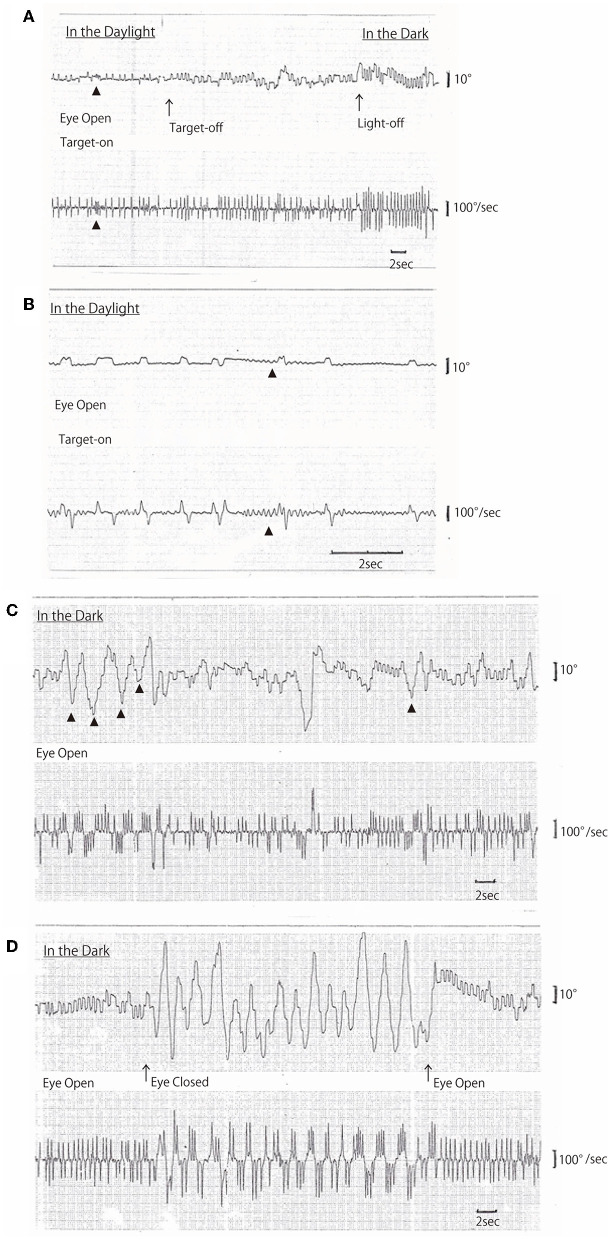
**(A**–**D)** Horizontal AC-electrooculogram (EOG) recordings. In each figure, the upper graph shows semi-eye position (time constant 3 s) and the lower graph shows eye speed (time constant 0.03 s). In each graph, the upper direction indicates rightward eye movement, and the lower direction indicates leftward. Chart speed is 5 mm/s in **(A**, **C, D)**, and 25 mm/s in **(B)**. **(A, D)** were continuously recorded. **(A)** Square wave jerks (SWJs) occurred in the following three conditions: first, in the daylight under the target-on state; second, under the target-off (arrow) state; and finally, light-off (arrow) in the dark. The amplitudes of SWJs were 4–5 deg under target-on, 5–6 deg under target-off, and 6–8 deg in the dark. The frequency of SWJs also gradually increased as follows: 1 Hz under target-on state, 1.5 Hz under target-off state, and 2 Hz in the dark. Ocular microflutters occurred among SWJs (arrowheads). **(B)** SWJs and ocular microflutter (arrowheads) occurred in the daylight under target-on state [recorded at 25 mm/s, i.e., as five times as in **(A)**]. **(C)** Macro SWJs (amplitude 5 deg) and a single or several saccadic ping-pong gaze (SPPG, arrowheads in the upper graph, amplitude 20–40 deg) occurred alternately in the dark with eyes open. **(D)** In the dark, macro SWJs (5–10 deg) occurred in a series with eyes open, and then with eyes closed, SPPG with high amplitude (70 deg) occurred continuously and macro SWJs disappeared. With eyes open again, macro SWJs reappeared continuously and SPPG disappeared.

**Figure 2:**
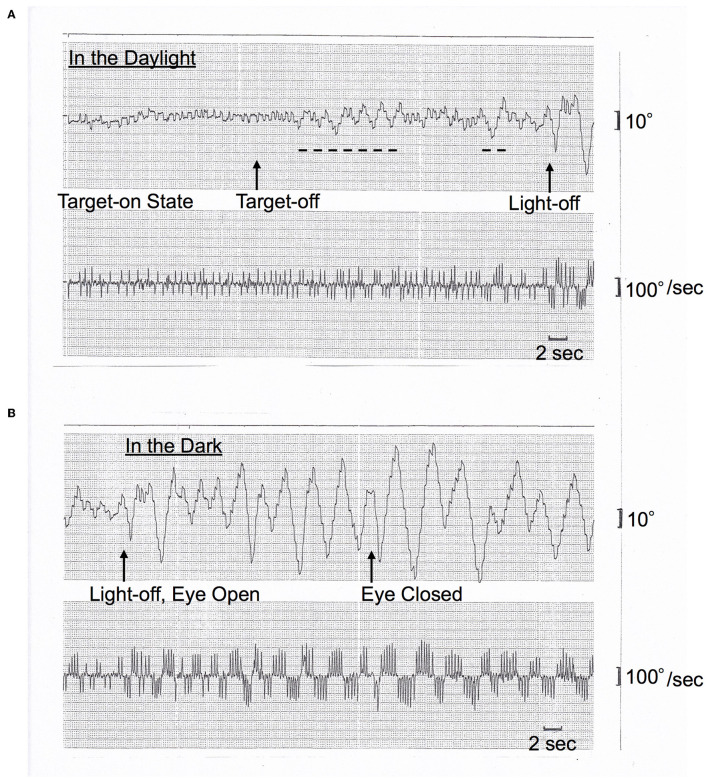
**(A**, **B)** Horizontal AC-EOG recordings. In each figure, the upper graph shows semi-eye position (time constant 3 s) and the lower graph shows eye speed (time constant 0.03 s). In each graph, the upper direction indicates rightward eye movement, and the lower direction indicates leftward. The chart speed is 5 mm/s. This figure was recorded one and a half years after Figure 1. **(A)** In the daylight, macro SWJs (5 deg) occurred under the target-on state from the start, and then under the target-off state, a single or several SPPGs with small amplitude (15–20 deg) occurred (dashed lines, 15 deg) alternately with macro SWJs. Subsequently, the light was turned off [**(A)** continues to **(B)**]. **(B)** In the dark, macro SWJs soon disappeared. Alternatively, SPPG appeared continuously and got larger (to 50 deg) than in the daylight. And then, with eyes closed, giant SPPG emerged with an amplitude of 70 deg, reaching the full range.

On AC-EOG examination, no decrease in range or velocity of voluntary vertical gaze was observed. However, horizontal macro SWJs (6–10) (around 5 deg in amplitude) occurred frequently (40–60/min) during fixation (Figures 1A, B, refer to Supplementary Video 1 by infrared CCD camera). SWJs are defined as “pairs of small horizontal saccades (typically <2 deg) that take the eye away from the primary position and then return it after 200–300 ms (6, 7),” and macro SWJs are defined as “large (>4 deg) SWJs (6, 7).” Along with SWJs, sometimes ocular microflutter (7, 9) of small amplitude (<2 deg) appeared with a saccade frequency of 8–10 Hz. This microflutter consisted of 5–10 back-to-back saccades with no intersaccadic interval (arrowheads in Figures 1A, B). SWJs occurred more frequently and largely under target-off state than under target-on (Figure 1A). In the dark, macro SWJs (5–10 deg) often occurred in a series (Figures 1A, C, D). In addition, with eyes open in the dark, there appeared either a single or several horizontal “saccadic ping-pong gaze” (SPPG) events with amplitude of 20–40 deg alternately with macro SWJs (arrowheads in Figure 1C, refer to Supplementary Video 2 by infrared CCD camera). With eye closure in the dark, SPPG with high amplitude (30–70 deg) appeared continuously and SWJs disappeared (Figure 1D). SPPG consisted of the following two components: (1) horizontal irregular pendular oscillations of the eyes (ping-pong gaze, PPG). Her eyes oscillated conjugately from the midpoint at a period of 2–4 s and amplitude of 20–70 deg, sometimes reaching the full range; and (2) alternating 4–8 small saccades per one period (refer to eye velocity traces in Figures 1C, D) superimposed on the pendular oscillations (saccadic ping-pong gaze, SPPG). She exhibited saccadic pursuit with normal amplitude when following a horizontal and vertical sinusoidal target (40 deg at a period of 3 s). SPPG in the dark was transiently suppressed by cold caloric stimulation (15°C, 20 mL). One and a half years after the first examination, along with her progression of postural instability, there appeared mild decrease in range and velocity of vertical gaze (10) (hypometric and stepwise saccades with peak velocity <200 deg/s and with saccade amplitude 20 deg). The fast phase of vertical optokinetic nystagmus also became slow or disappeared. In addition, SPPG was exacerbated. Even in the daylight, a single or several SPPG with small amplitude (15–20 deg) appeared under the target-off state (Figure 2A). In the dark with eyes open, SPPG at a period of 2–3 s emerged immediately and got larger, and then with eye closure, giant SPPG emerged with amplitude to 70 deg reaching the full range (Figure 2B, refer to Supplementary Video 2).

The atrophy of the midbrain and superior cerebellar peduncles was seen on MRI images (MR Parkinsonism Index (11, 12) 19.3) (Supplementary Figure 1). Mild striatal dopamine deficiency was found on dopamine transporter imaging (SBR Ave. 4.75, Z-score −2.60, Supplementary Figure 2). Elevated ^18^F-PM-PBB3 (^18^F-Florzolotau) binding, implying regional accumulation of tau fibrils, was observed in the brainstem, subthalamic nucleus, and basal ganglia by PET scanning (Supplementary Figure 3). Eventually the patient was diagnosed as having probable PSP by a combination of level 1 in oculomotor dysfunction (vertical supranuclear gaze palsy) and level 1 in postural instability according to the MDS criteria. She has been followed up in our outpatient clinic. Her postural instability has increased, and she needs her husband's help in almost all activities of daily living.

## Discussion

### Saccadic ping-pong gaze (SPPG) instead of transitory alternating saccades (TAS) in PSP

We described in detail a short-cycle, horizontal periodic alternating (ping-pong) gaze with superimposed small saccades, i.e., saccadic ping-pong gaze (SPPG) in a patient with PSP. This SPPG was usually observed in the dark and with eye closure by using EOG. The waveform of SPPG was somewhat similar to that of saccadic pursuit on a horizontal sinusoidal target. Our patient actually showed saccadic pursuit similar to SPPG on a horizontal sinusoidal target (40 deg at a period of 3 s). In Japan, this abnormal eye movement was first observed not in a patient in a coma but in a patient with spinocerebellar degeneration (SCD), in the dark with EOG, by Komatsuzaki and Mizutani (3). They described it as “horizontal alternating ocular deviation at a period of 3 s on which saccades superimpose” and named this phenomenon “transitory alternating saccades (TAS).” Since then, “TAS” has been reported in rare cases with PSP (13–15) in Japanese (not in English) literature. According to the reports with EOG in Japan, five patients with PSP showed TAS only in the dark and with eye closure at a period of 2–6 s and amplitude of 10–40 deg with alternating 4–10 small saccades (Table 1). In addition to TAS, SWJs corresponding to level 3 in oculomotor dysfunction of the MDS criteria were observed in four out of the five patients with PSP as well as the present patient. On the other hand, SPPG in comatose patients was described as a saccadic variant of “ping-pong gaze” (PPG) (1). PPG, short-cycle and horizontal periodic alternating gaze deviation, has been repeatedly described since Fisher (2) first documented it in 1967. The term PPG was first used by Senelick (16). The so-called TAS in PSP and SCD and SPPG in the present patient shows essentially the same waveform as SPPG in coma (1, 17). We think the term “transitory alternating saccades” does not express appropriately the features of this abnormal eye movement because the meaning of “transitory” is vague in this context. We suggest this eye movement be referred to as SPPG instead of TAS. SPPG in PSP in the dark can be differentiated from macro SWJs, macrosaccadic oscillations, ocular microflutter, opsoclonus, or acquired pendular nystagmus by using EOG.

**Table 1:** Clinical manifestation and features of so-called TAS (SPPG) in PSP.

		**Clinical manifestation**	**EOG findings of TAS**			
**No**	**References**	**Sex, age, duration**	**Amplitude (deg)**	**Period (sec)**	**No of small saccades**	**Occurrence condition**
1	Okuma et al. (13)	PSP	20–40	2–3	4–6	Not mentioned
		Male, 71 yo, 5 y				
2	Urabe et al. (14)	PSP	20	3	8–9	In the dark, Eye closed
		Male, 59 yo, 2 y				
3	Urabe et al. (14)	PSP	20–30	3–5	6–8	In the dark, Eye closed
		Male, 60 yo, 3 y				
4	Urabe et al. (14)	PSP	10–20	2–5	6–10	In the dark, Eye closed
		Female, 68 yo, 6 y				
5	Yokata et al. (15)	PSP, FTD	10–30	3–5	4–10	In the dark, Eye closed
		Male, 82 yo, 8 y				
6	Present case	PSP	15–70	2–4	4–8	In the dark, Eye closed
		Female, 63 yo, 5 y				

SCD, Spinocerebellar degeneration; PSP, Progressive supranuclear palsy; FTD, Frontotemporal dementia.

### Differences between SPPG in PSP and PPG/SPPG in coma

The conditions where SPPG in PSP appears are quite different from those where PPG/SPPG in coma does. PPG/SPPG in coma usually occurs more steadily and continuously than SPPG in PSP. PPG/SPPG in coma has higher amplitudes, described as “eyes constantly roved from one extreme lateral position to the other, every 2 s” (2). SPPG observed in our patient with PSP appeared most apparently in the dark and with eye closure. In addition, the degree of severity of SPPG changed according to different conditions. In the daylight under a target-off state and in the dark, a single or several SPPG with small amplitude occurred alternately with SWJs. Additionally, with eye closure in the dark, SPPG with high amplitude occurred continuously and SWJs disappeared. On the other hand, in patients in coma, no report is available that SWJs occurred alternately with SPPG. As already mentioned, PPG in coma has two types. One is PPG without saccadic intrusions and the other is saccadic PPG (SPPG). One patient in coma was reported to have shown the transition from PPG to SPPG (1), and Johkura et al. argued that SPPG was occurring in patients in a lighter state of consciousness and was possibly related to less extensive brain damage than PPG. PPG without saccadic intrusions has never been observed in conscious patients with PSP.

### The mechanism of SPPG in PSP

The mechanism of PPG/SPPG is not yet fully understood (17). PPG/SPPG is usually seen in unconscious patients with bilateral disconnection of the cerebrum from the midbrain, while the lower brainstem and cerebellum remain largely intact (17, 18). In addition to this disconnection, other factors are probably involved in PPG/SPPG. Senelick argued that a small hemorrhage found in the midline deep vermis was thought to have disrupted the neural integrator that holds the position of gaze, resulting in PPG (16). In unresponsive patients, drift-back of eyes after head rotation (oculocephalic response) was found to be much more rapid than that in healthy, alert controls, and the authors argued that the finding implied dysfunction of the brainstem reticular and, possibly, cerebellar connections (19). Here, we propose that PSP, a neuro-degenerative disorder, should be added as one of the etiologies of SPPG. We assume that SPPG in PSP results basically from serious pathological changes in the midbrain and basal ganglia (20) and visual deprivation in the dark and with eye closure. This assumed cause of SPPG *in PSP* is somewhat similar to that of PPG/SPPG *in coma* in the light of bilateral disconnection of the cerebrum from the midbrain with visual deprivation. In addition, in PSP, steady fixation is disrupted by macro SWJs. SWJs also occur in normal subjects, but in PSP, they are larger and more frequent, which is possibly attributable to the changes in the cerebellum and its output through the superior cerebellar peduncles (8–10). SPPG in the present patient with PSP appeared alternately with frequent macro SWJs in the dark, and with eye closure, giant SPPG appeared continuously and SWJs disappeared. The present patient showed saccadic pursuit on a horizontal sinusoidal target, which was similar to her own SPPG. Her SPPG in the dark was transiently suppressed by cold caloric stimulation. These findings suggest that SPPG in PSP emerges if the following three conditions are met: (1) midbrain and basal ganglia pathology, as we observed in MRI and tau PET, (2) visual deprivation in the dark, and (3) cerebellar dysfunctions, especially those of cerebellar oculomotor systems, including oculomotor vermis (VI, VII) and the underlying caudal fastigial nuclei (fastigial oculomotor region), which generate both smooth pursuit and saccade (21, 22). In addition, we think that predominant postural instability with a broad-based stance in this patient is also associated with midline cerebellar dysfunction.

## Data availability statement

The original contributions presented in the study are included in the article/Supplementary material, further inquiries can be directed to the corresponding authors.

## Ethics statement

The studies involving human participants were reviewed and approved by the Medical Research Ethics Committee at Tokyo Rinkai Hospital. The patients/participants provided their written informed consent to participate in this study. Written informed consent was obtained from the individual(s) for the publication of any potentially identifiable images or data included in this article.

## Author contributions

HN and HI: conception, organization, execution, and writing the draft and figure. TK and AInos: conception and execution. TH, HS, TM, and YM: organization and execution. YT and HE: execution and writing the figure. AInom and SM: execution. All authors contributed to the article and approved the submitted version.
