# Supplementary material for: Case report: Saccadic ping-pong gaze in progressive supranuclear palsy with predominant postural instability
Source: Front Neurol. 2023 Mar 1;14:1100931. doi: 10.3389/fneur.2023.1100931 (PMC10014735; doi:10.3389/fneur.2023.1100931)
Supplement: Supplementary file 3 [file Data_Sheet_1.docx]

**Supplementary Videos**

**Supplementary Video 1**

Abnormal eye movements recorded by infrared CCD camera

1) Square wave jerks (SWJs): Horizontal SWJs occur frequently.

2) Saccadic ping-pong gaze (SPPG) in total darkness: Horizontal irregular large SPPG occurs almost continuously in total darkness.

**Supplementary Video 2**

Postural instability in standing and gait

1) Standing (closing eyes): Her stance was wide based. When she was asked to close her eyes, she fell backward.

2) Gait (Timed Up and Go test): She needed to push the arms of a chair when she tried to stand up. She was not able to stand up smoothly because her center of gravity was located posteriorly in her base of support. She managed to stand up with the hip in hyperflexion. She walked with wide-based and short-stepped. Her gait was characterized by reduced rotation of the trunk. She turned “en bloc”.

3) Gait (Supporting thorax from behind): When she was supported just below her both armpits from behind, she was able to stand up easily, walk straight and turn a little smoothly.

4) Gait (Touching paraspinal region): In the gait assessment immediately after the one noted above, she was able to walk straight just by being touched on the thoracic paraspinal region like using a sensory trick in dystonia.

5) Gait (Transverse Lines): The lines to step over as visual cues were not effective for her to walk smoothly.

**Supplementary Figures**

**Supplementary Fig. 1**

T1-weighted Magnetic resonance (MR) images of midbrain and pons (**A**), middle cerebellar peduncle (MCP) (**B**) and superior cerebellar peduncle (SCP) (**C**) in the present patient. The following values were measured on these images: midsagittal area of the midbrain (area M) and that of pons (area P), width of the MCP in sagittal plane (width MCP), and width of the SCP in coronal plane (width SCP). The MR Parkinsonism Index (MRPI) was calculated with these values as follows: MRPI = (area P / area M) × (width MCP / width SCP)^10^. MRPI of the present case is 19.3 (normal range 6-13, PSP range 13-29)^11^.

**Supplementary Fig. 2**

Dopamine transporter imaging shows bilateral mild striatal dopamine deficiency (SBR Ave. 4.75, Z-score –2.60). Age-matched normal SBR score is 8.24 (average) and 1.35 (1 SD). SBR score of -2SD from average is 5.54. SBR, specific binding ratio. From color imaging, dopamine deficiency appears to be predominant in putamen.

**Supplementary Fig. 3**

Tau PET with PM-PBB3 compared to cognitively healthy elderly, presented case showed a remarkable uptake of PM-PBB3 in the brainstem, subthalamic nucleus, and basal ganglia, which is typically observed in patients with PSP (yellow arrows). In contrast, rostral interstitial nucleus of the medial longitudinal fasciculus showed relatively slight uptake of PM-PBB3. Since PSP patients occasionally have tau pathology even in cerebellum, setting the reference region for PET to the cerebellum may underestimate the accumulation of tau PET ligand. Therefore, to avoid such underestimation^X^, radiotracer binding was calculated as SUVR using an optimized reference tissue method instead of cerebellar grey matter reference. PSP, progressive supranuclear palsy. SUVR, standardized uptake value ratio. White arrow heads indicate off-target binding of PM-PBB3 to the choroid plexus.

Reference X. Tagai K, *et al*. An optimized reference tissue method for quantification of tau protein depositions in diverse neurodegenerative disorders by PET with ^18^F-PM-PBB3 (^18^F-APN-1607). MedRxiv. doi: <https://doi.org/10.1101/2022.02.13.22270135>
