# Supplementary figures and images for: Case report: Saccadic ping-pong gaze in progressive supranuclear palsy with predominant postural instability
Source: Front Neurol. 2023 Mar 1;14:1100931. doi: 10.3389/fneur.2023.1100931 (PMC10014735; doi:10.3389/fneur.2023.1100931)

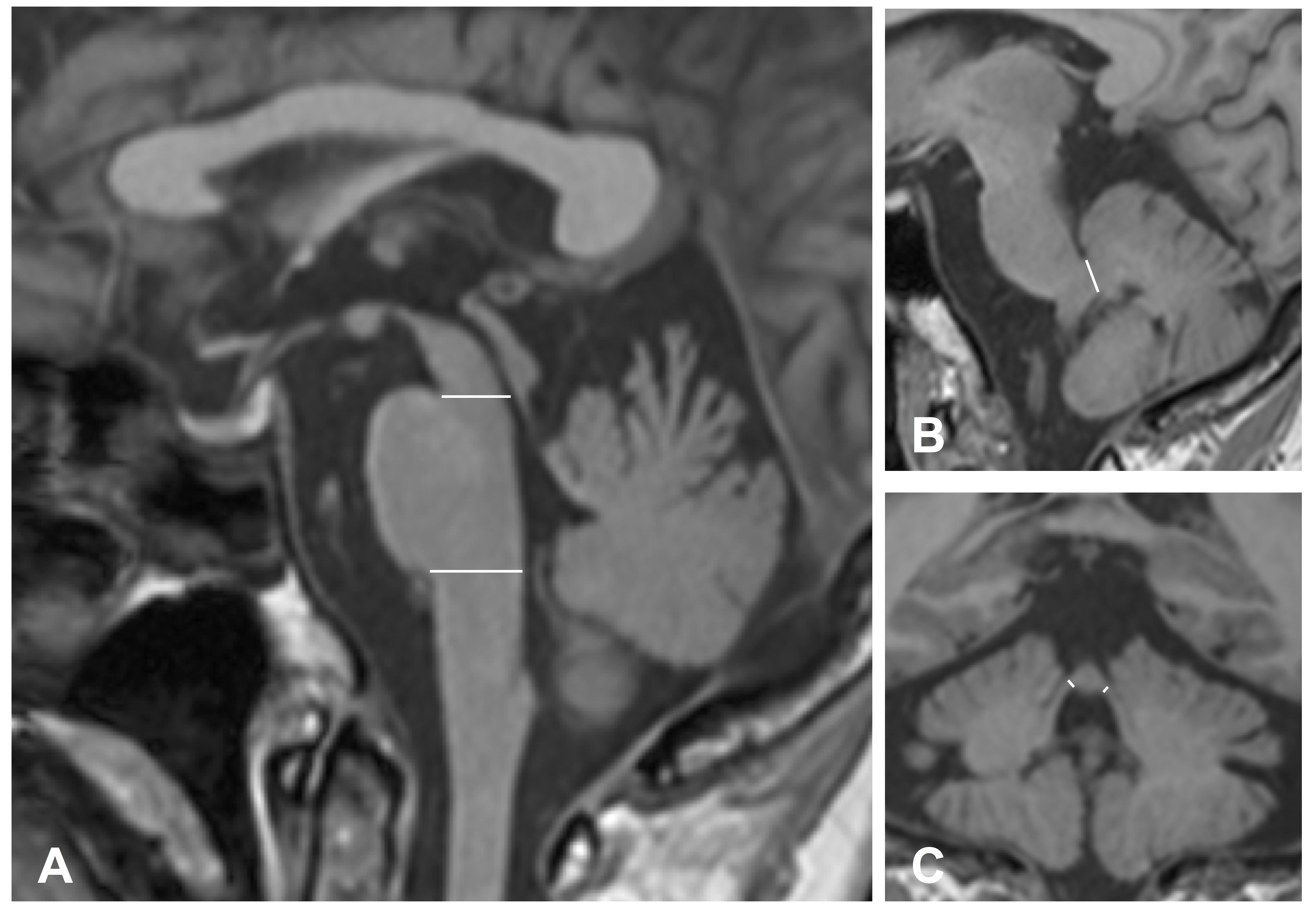

Supplement: Supplementary file 4 [file Image_1.JPEG]

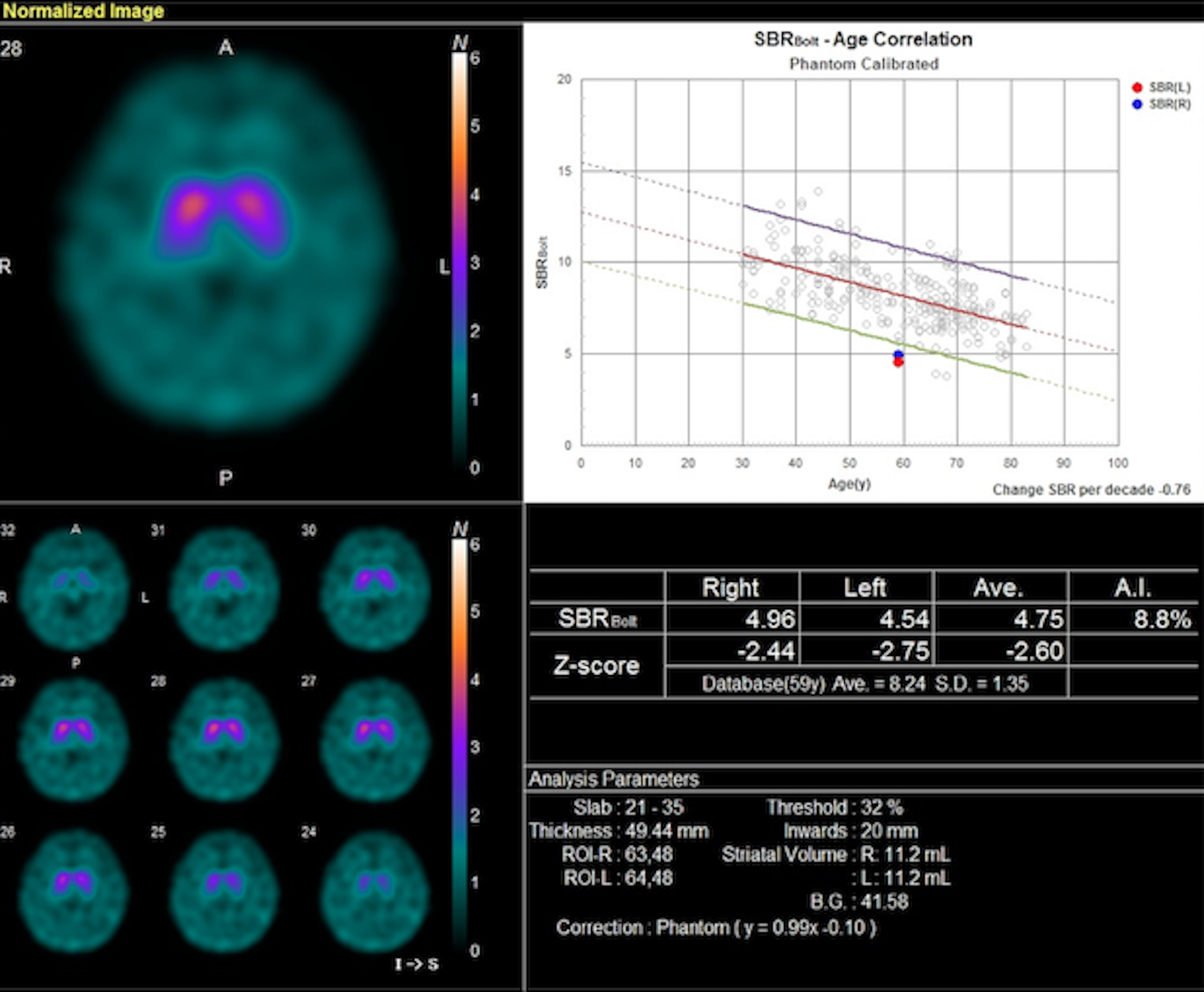

Supplement: Supplementary file 5 [file Image_2.JPEG]

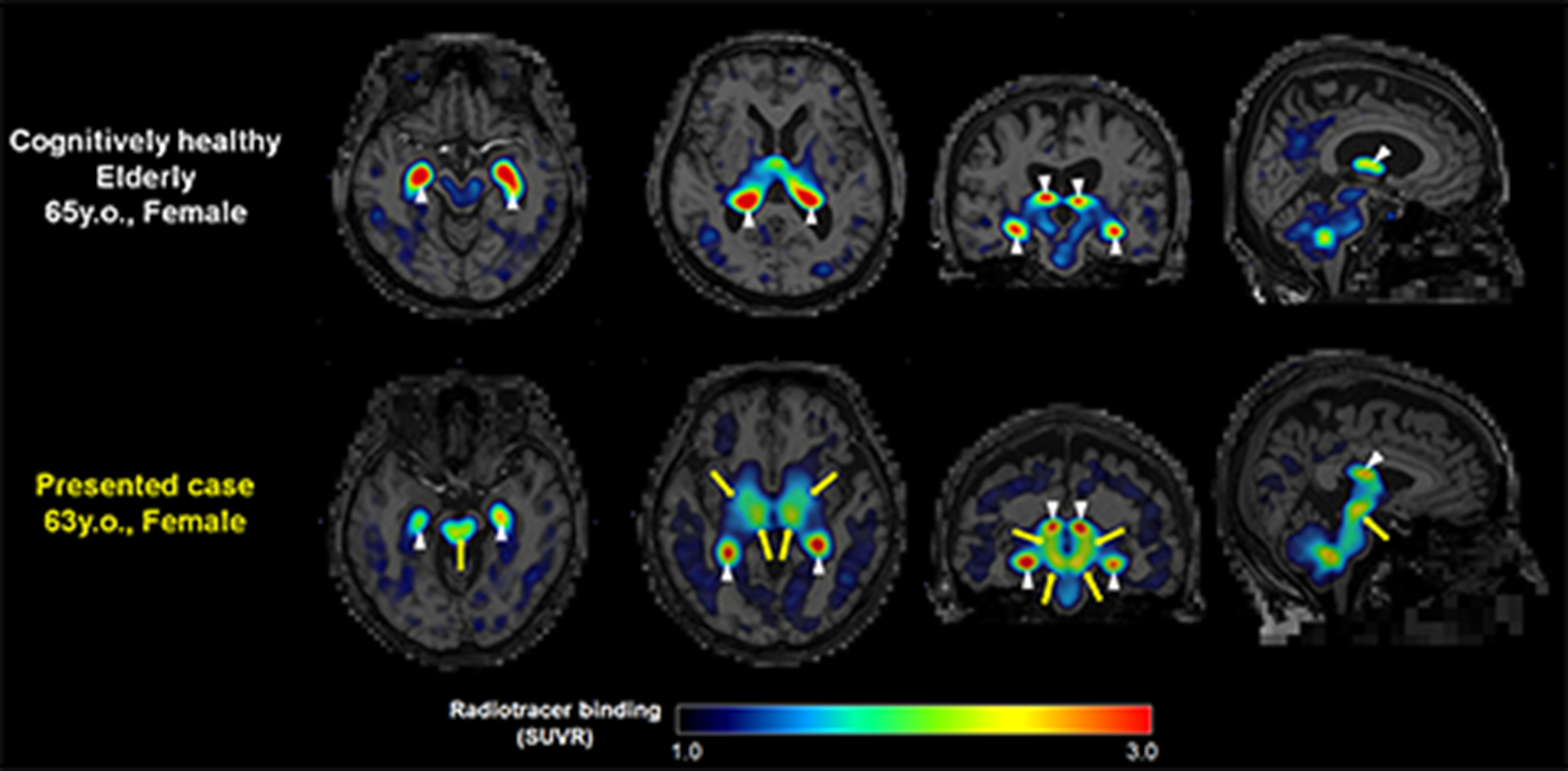

Supplement: Supplementary file 6 [file Image_3.PNG]
